# Agricultural biotechnology in the courts: judicial opinions and commentary

**DOI:** 10.3389/fbioe.2025.1592675

**Published:** 2025-08-11

**Authors:** Drew L. Kershen

**Affiliations:** Earl Sneed Centennial Professor of Law Emeritus, University of Oklahoma, Norman, OK, United States

**Keywords:** agricultural biotechnology, breeding, environment, human health, litigation, judicial opinions, plant scientists

## Abstract

Seven jurisdictions from around the world have issued judicial opinions that address fundamental issues about the governance and regulatory systems of agricultural biotechnology. This article summarizes these legal proceedings and describes their impact upon agricultural biotechnology. The article then provides a commentary and critique of the legal proceedings and resulting judicial opinions.

## Introduction

Many courts around the world have issued judicial opinions about agricultural biotechnology. This author considers seven opinions, from seven different jurisdictions, as the most significant and consequential. In chronological order of issuing the opinion, these seven jurisdictions are New Zealand, the European Union, Kenya, Ghana, the Philippines, South Africa, and the United States. Each of these opinions decided whether modern techniques of molecular biology would be allowed or disallowed for crop breeding and crop improvement within that jurisdiction. These opinions also affect agricultural trade, though trade is not a significant focus of this article. Effectively, these opinions were deciding the future of agriculture for that jurisdiction. More broadly, each of these opinions is an important influence for the global debate about the future of agriculture.

In Part One of this article, the author presents a summary of the litigation and judicial opinions. The author then provides an impact analysis of these opinions for going forward with agricultural biotechnology in that jurisdiction. Finally, the author explains the present status, as of May 2025, of agricultural biotechnology and its food and feed products for each of these seven jurisdictions.

In Part Two, the author writes a commentary about these judicial opinions. The author critiques each of these judicial opinions and explores, from his pro agricultural biotechnology perspective, the deeper meanings and implications of these judicial opinions.

## Part One: seven jurisdictions: litigation summary, impact, and present status

### New Zealand

In October 2012, Scion, the Crown Research Institute for forest resources, applied to the Environmental Protection Agency (EPA) for a determination that forest plants created using Zinc-Finger Nuclease Type 1 (ZFN-1) and Transcription Activator-Like Effector Nucleases (TALENS) were exempt from the Hazardous Substances and New Organisms Act of 1996 (HSNO 1996). Section 2 of HSNO 1996 included genetically modified organisms as new organisms subject to the 1996 Act. Section 3 of HSNO 1996 gives a list of organisms that are not genetically modified and thus not subject to HSNO and the implementing EPA regulations. Among those organisms exempted included organisms from “chemical or radiation” treatments (chemical or radiation mutagenesis). Scion argued that ZFN-1 and TALENS were similar to (equivalent to) chemical mutagenesis and, as set forth in HSNO Section 3 and the EPA regulations, created organisms not genetically modified.

After considering information supplied by Scion, considering objections to the application supplied by the Sustainability Council of New Zealand Trust, and after carefully reading the language of HSNO 1996, the EPA staff recommended to the EPA’s governing HSNO Section 26 Committee that Scion’s application be denied. [The Section 26 Committee is the deciding authority under HSNO 1996.]. The EPA staff focused on the facts that these two techniques used *in vitro*, laboratory, modern molecular biology to make genetic changes. In the opinion of the EPA staff, these facts made Scion’s forest plants “new organisms” subject to the HSNO 1996 law and implementing regulations.

The EPA’s governing Section 26 Committee considered the same information as the staff and the staff’s recommendation. The Section 26 Committee decided that ZFN-1 and TALENS were similar to chemical mutagenesis. In fact, the Section 26 Committee determined that the mutations from ZFN-1 and TALENS would be indistinguishable from mutations occurring naturally or from chemical mutagenesis. The governing Committee concluded that Scion’s forest plants thereby came within Section 3 exemption to HSNO 1996. Consequently, Scion would be able to conduct field trials and, ultimately, to release its forest plants into New Zealand without being required to pass through the strict regulatory regime HSNO 1996 established.

The Sustainability Council sought judicial review of the Section 26 Committee’s decision by filing a lawsuit in the High Court, the trial court level, of New Zealand (Wellington Registry). The case name is *The Sustainability Council of New Zealand Trust against The Environmental Protection Authority* (Kershen, 2015).

In its opinion (The Sustainability Council, 2014), the High Court carefully and extensively discussed the technology issues and the various techniques for causing genetic mutations in plants. When the High Court finished this plant-breeding discussion, the High Court turned to competing interpretations of the statute (HSNO 1996) and the EPA regulations implementing the statute. The High Court’s decision can best be summarized by quoting from two paragraphs in the opinion.

“[66] A common feature of each of the techniques [the Section 3 list of exempt organisms] is that they were in common use at the time of the regulations. It makes sense that the regulations would exempt techniques that were well understood and established. There is less likely to be scientific and technical uncertainty about the effects of such techniques. It would therefore be consistent with the Act’s purpose and the precautionary principle to exempt such common techniques rather than subjecting them to the approval regime of the Act. … it would follow that new techniques … were not intended to be included in the exemption.”

And the quotation from the second paragraph is:

“[73] For all these reasons, I consider the correct interpretation is that put forward by the Sustainability Council. That is, reg 3(1)(b) exempts organisms that are generated from organs, tissues or cell culture using any of the following techniques: selection and propagation of somaclonal variants, embryo rescue, cell fusion (including protoplast fusion), and chemical or radiation treatments that cause changes in chromosome number or cause chromosome rearrangements. It follows that the Authority [i.e., the Section 26 Committee] erred in its interpretation of the regulation because it considered that the regulations did not set out an exhaustive list and that techniques that are comparable and sufficiently similar to those listed in the Regulations should also be excluded.”

The High Court interpreted Section 3 of HSNO 1996 as stating an exhaustive list of exempt organisms. As ZFN-1 and TALENS are not on the Section 3 exhaustive list of exempt organisms, organisms created by these unlisted techniques are subject to HSNO 1996. The High Court therefore granted relief to the Sustainability Counsel and rejected Scion’s application. Under the High Court’s opinion, Scions plants must pass through the strict regulatory regime HSNO 1996 established.

There was no appeal from the High Court decision, making it binding upon the legal system of New Zealand.

The detrimental impact upon agricultural biotechnology was immediate. After 2014, initiation of field trials of plants created through modern molecular biology (e.g., gene-editing techniques) ceased. Plants created through modern molecular biology do not exist in the agricultural sector of New Zealand because they are illegal and prohibited unless these plants have passed through the stringent regulatory procedures the HSNO 1996 established. As far as the author knows, since 2014, no plant breeder has even applied to the EPA for approval for a field trial or commercial release because the process for gaining approval is so burdensome and the likelihood of success is so small as to make the effort not worthwhile (USDA-GAIN NZ, 2023).

By contrast, Food Standards Australia New Zealand (FSANZ) has approved many food ingredients from genetically modified and gene-edited crops. While these food ingredients are listed on product labels as genetically modified, the author understands that New Zealand food stores carry a goodly number of these FSANZ-approved foodstuffs and that consumers calmly purchase these foodstuffs. Animal feeds made from genetically engineered and gene-edited crops are regularly and widely imported and commonly used by animal agriculture (USDA-GAIN NZ, 2023).

In New Zealand, the way-forward for agricultural biotechnology is through legislative and regulatory action. In December 2024, the Government of New Zealand introduced to Parliament a Gene Technology Bill. The Government says the objective of the Bill is to establish a new regulatory regime for genetically modified and gene-edited organisms that is: risk-proportionate (tiered regulatory oversight based on product risk, not process); efficient in application and timely in decision-making; flexible to accommodate technological and policy developments; and aligned with international and Maori obligations. As of May 2025, the Health Committee of the NZ Parliament is holding public hearings on the Gene Technology Bill (Gene Technology, 2024). Significant legislative actions remain to occur before, if ever, the Gene Technology Bill would become the law of New Zealand.

### The European Union

On 29 July 2013, Confédération paysanne, a French agricultural union, joined by eight other French environmental organizations, asked the French Minister of Agriculture to oppose the development of herbicide-tolerant crops. When the Minister of Agriculture did not satisfy their request, Confédération paysanne formally wrote the French Prime Minister, in December 2014, to ban the cultivation and marketing of herbicide tolerant (HT) canola varieties. These HT canola varieties came into existence through plant breeding techniques that are grouped together under an umbrella term of new genomic techniques (NGTs). When the Prime Minister declined to do so, on 12 March 2015, Confédération paysanne applied to the Council of State, France for a court order mandating the Prime Minister to ban these NGTs canola varieties (Confédération paysanne timeline, 2015). (The Council of State, France is the highest court for administrative justice within the French legal/judicial system.).

Due to the importance of the case for European Law, the Council of State, France referred the legal matter to the Court of Justice European Union (CJEU). [Bobek, 2018, ¶¶ 24–37]. This referral resulted in the CJEU docketing the first legal case in the EU related to gene-edited plants: Confédération paysanne against Prime Minister (2018) [Confédération paysanne 2018].

After docketing and under the procedures of the CJEU, the Court does not act until it receives an advisory opinion from the Office of the Advocate General about how to resolve the legal questions before the Court. Advocate General Bobek issued the advisory opinion on 18 January 2018 (Bobek, 2018).

In the advisory opinion, Advocate General Bobek opined that undefined terms, such as “mutagenesis” in the EU Directive 2001/18/EC, must be dynamic in interpretation so that the law can properly react to technical and social evolution. Otherwise, AG Bobek wrote,

“The suggestion that the interpretation of such notions ought to be ‘frozen’ in the factual or societal circumstances that prevailed when those notions were passed into law would represent a singularly *originalist* approach to legal interpretation, not frequently encountered on this side of the Atlantic.” [Bobek, 2018, ¶ 100, italics in the original.]

Based on this dynamic interpretative approach, AG Bobek concluded,

“The exemption laid down in Article 3(1) of Directive 2001/18, read in conjunction with its Annex IB, covers all organisms obtained by any technique of mutagenesis, irrespective of their use at the date of the adoption of that directive, on the condition that they do not involve the use of recombinant nucleic acid molecules or genetically modified organisms other than those produced by one or more of the methods listed in Annex IB.” [Bobek, 2018, ¶168].

On 25 July 2018, the CJEU issued the first judicial opinion in the EU on the legal status of gene-edited plants. The CJEU rejected the advisory opinion of AG Bobek and adopted an originalist approach to the interpretation of Directive 2001/18. Confédération paysanne against Prime Minister (2018) [Confédération paysanne 2018]. Quoting from the judicial opinion, the CJEU decided:

“[51] … Article 3(1) of Directive 2001/18, read in conjunction with point 1 of Annex IB to that directive, cannot be interpreted as excluding, from the scope of the directive, organisms obtained by means of new techniques/methods of mutagenesis, which have appeared or have been mostly developed since Directive 2001/18 was adopted. Such an interpretation would fail to have regard to the intention of the EU legislature, reflected in recital 17 of the directive, to exclude from the scope of the directive only organisms obtained by means of techniques/methods which have conventionally been used in a number of applications and have a long safety record.

“[52] That finding is supported by the objective of Directive 2001/18, as is apparent from Article 1 thereof, in accordance with the precautionary principle, to protect human health and the environment. …” [Confédération paysanne 2018, ¶¶ cited in the text quotations].

After the CJEU decision of 2018, the case returned to the Council of State, France to determine which specifically-identified mutagenic techniques (first) have been conventionally used in a number of breeding applications and (second) have a long safety record of use prior to the year 2001. On 7 February 2020, the Council of State, France ordered and decided,

“…ordered the Prime Minister to determine … the restrictive list of techniques or methods of mutagenesis which have conventionally been used and have a long safety record. The Counsel of State considered, in [the 7 February 2020 decision], that both the techniques or methods of ‘directed mutagenesis’ or ‘genome edition’ and the techniques of ‘*in vitro*’ random mutagenesis appeared or were mainly developed after the date on which Directive 2001/18 was adopted and must therefore be considered to be subject to the obligations imposed on GMOs by that directive …” [Szpunar, 2022, ¶16].

Thus, in its 7 February 2020 decision, the Council of State, France ruled that three mutagenic techniques (directed mutagenesis, gene-editing, and *“in vitro”*) are subject to the EU Directive 2001/18. By contrast, the Council of State also ruled that *“in vivo”* techniques are conventional techniques with a long history of safety and, thus, outside of the regulation EU Directive 2001/18.

The Council of State, France notified its decision to distinguish “*in vivo*” mutagenesis from “*in vitro”* mutagenesis to the EU Commission. [Szpunar, 2022, ¶18]. This notification gave rise to the second EU judicial opinion related to mutagenic plant breeding techniques. The second case is Confédération paysanne against Prime Minister, 2023 [Confédération paysanne 2023].

In accordance with standard procedures, the CJEU did not act until it had received an advisory opinion from the Advocate General. Advocate General Szpunar issued the advisory opinion on 27 October 2022. In the advisory opinion, Advocate General Szpunar pointed out that the EU Commission, based on advice from the European Food Safety Authority (EFSA) and other Scientific Committees, dissented from the Council of State’s distinction between “*in vivo*” mutagenesis and “*in vitro*” mutagenesis because the molecular mechanisms are the same, the types of mutations are the same, and “*in vitro*” techniques have been used since the 1960s and widely used since the 1990s [Szpunar, 2022, ¶¶ 45–56]. Based on these facts, AG Szpunar advised that CJEU should rule that random mutagenesis “*in vitro*” should be legally the same as random mutagenesis “*in vivo*” and, therefore, exempt from Directive 2001/18 related to GMOs. [Szpunar, 2022, ¶ 70].

On 7 February 2023, the CJEU ruled that “*in vitro*” random mutagenesis is exempted from the GMO Directive 2001/18 because this technique has conventionally been used in many breeding applications prior to 2001 and had a long safety record with regard to its use. The CJEU 2023 opinion rejected the Council of State, France distinction between “*in vivo*” mutagenesis and “*in vitro*” mutagenesis. [Confédération paysanne 2023, Rule after ¶ 67]. The CJEU ruled that “*in vivo*” and “*in vitro*” breeding techniques are legally equivalent. As legally equivalent, the CJEU decreed that both *“in vivo”* and *“in vitro”* breeding techniques are not regulated by EU Directive 2001/18. The reader should note that the CJEU and Advocate General Szpunar disagreed with the Council of State’s factual statements, in its 7 February 2020 opinion, about *“in vitro”* techniques being of recent development and usage (i.e., after the year 2001).

In summary, the CJEU opinions of 2018 and 2023 make the following distinctions for EU law and regulations. First, genetically modified plants and gene-edited plants are considered post-2001 breeding techniques that are subject to the EU Directive 2001/18. Second, classical chemical or radiation mutagenesis, whether accomplished *in vivo* or *in vitro*, are considered conventional pre-2001 breeding techniques and, therefore, not subject to EU Directive 2001–18.

The impact of the two *Confédération paysanne* cases is to do exactly what AG Bobek foresaw in his 2018 Advisory Opinion. The EU regulatory regime related to modern plant breeding is frozen in time (pre-2001 versus post-2001) and prohibits genetically modified plants and gene-edited plants from being in commercial use in Europe unless plant breeders gain authorization through the stringent requirement of Directive 2001/18. In reality, plant breeders have rarely even tried to gain approval. Except for one transgenic crop, approved under EU law in 1998 prior to Directive 2001/18, there are no genetically modified or gene-edited crops grown in European agriculture. Field testing is basically non-existent in Europe. When plant breeders do attempt field tests, activist vandals destroy the test plots (Negri, 2024).

EU Directive 2001/18, the focus of the judicial opinions explained above, relates to the release of crops into the environment (i.e., farmers’ fields in the EU). A separate and distinct Regulation (EU) No. 1829/2003 governs approval for food and feed uses of products consisting, containing, or derived from genetically modified organisms, regardless of whether they have been imported or not. Under the authority of Regulation (EU) No. 1829/2003, the EU Commission has authorized more than seventy foods and feeds from genetically engineered and gene-edited crops for the EU consumer markets. European animal agriculture uses significant quantities of GMO feedstuffs and would not be able to satisfy consumer demand for animal products without these GMO feedstuffs [USDA GAIN EU, 2023, *passim*]. By contrast, European food companies and grocers have been very reluctant to sell (as a practical matter, almost banned) foodstuffs containing ingredients from genetically modified or gene-edited crops from their inventory and grocery shelves.

The way forward for modern (post-2001) agricultural biotechnology is through legislative and regulatory action. In July 2023, the European Commission (EC) presented a proposal to the Council of the EU (the member states) to amend EU laws and regulations related to crops developed by the use of NGTs. In March 2025, the Council of Europe reached agreement on a common position related to the July 2023 EC proposal. By agreeing to a common position, trilateral negotiations between the Council, the EC, and the EU Parliament can begin about what amendments specifically will be made, if any, to the EU laws and regulations related to gene-edited plants. As of May 2025, this trilateral negotiation has just begun and is expected to take approximately 2 years (Morrison Forester, 2025).

In the political and cultural climate that presently exists in Europe, it is unlikely that agricultural biotechnology will be accepted any time soon. While the EC and the Council have proposed changes in the EU legal regime related to agricultural biotechnology, even these recent proposals face significant opposition. The European deadlock about agricultural biotechnology apparently has no foreseeable end (Brookes and Smyth, 2024).

### Kenya

On 8 November 2012, the Cabinet of the Republic of Kenya directed the Minister of Health to use Kenyan health and sanitation laws to prohibit the importation of food and feed from genetically modified crops and to prohibit the cultivation within Kenya of genetically modified crops. This Cabinet decision controlled until 3 October 2022 when the Cabinet of the Republic of Kenya lifted the 2012 prohibition. In 2022, the Cabinet allowed the National Biosafety Authority to use and execute Kenyan laws and regulations, that had been adopted in earlier years, to authorize importation and cultivation of genetically modified crops, food, and feed. Due to this 2022 Cabinet order, under previous decisions of the National Biosafety Authority, Kenyans could import and grow genetically modified white maize (corn) [Kenya Peasants League, 2025, ¶¶ 1& 5].

Soon thereafter, on 16 January 2023, the Law Society of Kenya (Law Society) filed a petition with the Environment and Land Court (E&L Court) at Nairobi. In the petition, the Law Society asked for a declaration by the E&L Court that the Attorney General (that is, the Government of Kenya [GOK]) had violated the rights of the Law Society and the Kenyan public to a clean and healthy environment. The Law Society also asked the E&L Court to mandate that the GOK be prohibited from allowing further cultivation, importation and exportation of genetically engineered maize and other requested relief. By this lawsuit, the Law Society was challenging the Cabinet’s 2022 decree lifting the 2012 ban on genetically modified crops, food, and feed. The case name is: *Law Society of Kenya v. Attorney General* [Law Society, 2023, ¶ 1 Judgment.]

After a thorough and extensive presentation of the submissions and legal arguments of the Law Society and the Attorney General and the relevant legal authorities domestically and internationally, the Justice of the E&L Court gave his analysis and determinations. To understand the decision, the best approach is to quote various paragraphs from the E&L Court judgment.

On 12 October 2023, the E&L Justice wrote,

“[298] What the court is called upon to determine is the effectiveness of the existing laws and regulations in as far as identifying, mitigating and addressing potential risks taking into account key environmental principles and concerns, and in particular the precautionary principle in sustainable development.

“…

“[330] It is not clear to this court why all these approvals [prior GOK approvals of GMOs for cultivation and import as food/feed] have not been challenged by the Petitioner [Law Society] if indeed there is a concern that GM foods pose a serious risk to the environment and human health.

“[331] In conclusion, it is the finding of this court that the Petitioner has not challenged the constitutionality of the laws governing GMOs, both international and domestic. The regulatory barriers that govern importation and cultivation of GMOs remain in force, and the same are presumed to be constitutional until the contrary is proven.

“…

“[337] The existing legal and institutional framework has been set up for the rigorous evaluation of GM organisms and GM foods relative to both human health and the environment. The evidence before the court shows that the National Biosafety Authority and other Agencies have the capacity in the identification of foods that should be subject to risk assessment and recommend appropriate approaches to safety assessment.

“…

“[339] Let me end by stating that as a country, we need to trust the institutions that we have in place, and call them to order in the event they breach the law. The *Biosafety Act* stipulates that the National Biosafety Authority should work in close collaboration with the Department of Public Health, which safeguards the health of consumers through food safety and quality control, surveillance, prevention and control of food borne diseases.

“…

“[342] With all these institutions, we should be confident that our health and environment is in good hands. It cannot be true they have all conspired to expose the rest of the population to the calamities alluded to in the Petition.

“[343] This court has not been shown any evidence to show that the Respondents [GOK] and the institutions named in the preceding paragraphs have breached the laws, regulations and guidelines pertaining to GM food, and in particular the approval of the release in the environment, cultivation, importation and exportation of Bt maize.

“[344] For those reasons, the petition dated 16 January 2023 is dismissed…” [Law Society, 2023, ¶¶ of Judgment given in the text quotations.]

Opponents of agricultural biotechnology did not passively accept the Judgment of the E&L Court. The opponents previously had filed three additional petitions (dated 18 September 2015, 13 October 2022, and 22 November 2022) against the GOK for authorizing the cultivation, importation and exportation of genetically modified crops. For these additional petitions, the opponents filed in the High Court of Constitutional and Human Rights at Nairobi. These petitions gave rise to a second case, as the High Court consolidated the three petitions into one proceeding: *Mwangi v. Attorney General* (2024) [Mwangi, 2024, ¶¶ 3–5].

On 7 November 2024, the High Court of Constitutional and Human Rights (High Court C&HR) rendered a ruling in this second case that never reached the substantive legal arguments and issues. Rather, the High Court C&HR immediately took notice that a court of equal status (the E&L Court) had already rendered a judgment on what appeared to be the same substantive legal arguments and issues. Thus, the High Court C&HR instructed Mwangi and the Attorney General to develop an analysis of whether the E&L Court decision was final and binding on the High Court C&HR so as to preclude continuing litigation and re-litigation. In legal terms, the High Court C&HR wanted to know about *res judicata* and *collateral estoppel*, which are legal terms that relate to the ability of parties in a dispute to relitigate decisions. [Black’s Law Dictionary].

The High Court C&HR decision can best be understood by reading several paragraphs of its opinion;

“[72] It is manifest that the Environment and Land Court judgment largely focused on the validity of the (GOK) decision to lift the ban of GMO foods in Kenya. That was the substratum of the case before the Environment and Land Court which considered the implication of lifting the ban on GMO foods in relation to the importation and exportation of Bt maize.

“[73] In arriving at its decision ELC made findings on a host of issues that were raised in the case. A significant determination that was made was on the safety of GMOs. The Court made a finding that the laws and regulations in place both nationally and internationally are proper and were made in a manner that guards against violation of fundamental rights such as protection of the right to a clean and healthy environment. The Court also noted that the laws were in harmony with the precautionary principle as interpreted by the Courts.

“[74] Additionally, it was observed that the (Law Society) had failed to challenge the constitutionality of the laws that govern GMOs and hence those laws enjoy the presumption of constitutionality until proven otherwise. …

“…

“[81] In view of the foregoing reasons, it is the finding of this Court that the current consolidated Petition is *res judicata*. The Court would be regurgitating the same issues that were exhaustively dealt with by the ELC Court if it were to insist on hearing the consolidated Petition. I hereby strike out the same with no order as to costs.” [Mwangi, 2024, ¶¶ of Ruling given in the text quotations.]

The Kenya Peasants League (KPL) appealed the decision of the High Court C&HR to the Kenya Court of Appeal. On 7 March 2025, the Court of Appeal ruled that the KPL had the legal right to appeal the High Court C&HR decision. The Court of Appeal made it clear that this 2025 appeal was only on the issue of whether the High Court C&HR had correctly ruled about the doctrine of *res judicata*. In other words, the Court of Appeal, by allowing the appeal, was only addressing the procedural issue about relitigation. The Court of Appeal made it clear that the underlying substantive issues, relating to constitutional issues about a clean and healthy environment, are not part of this 2025 appeal. The Court of Appeal docketed the hearing on the 2025 relitigation appeal for the second term of 2025. In Kenya, the second court term begins in late April 2025 [Kenya Peasants League, 2025, ¶¶ 50–53].

By these three decisions (Law Society, 2023; Mwangi, 2024; Kenya Peasants League, 2025), the alternative paths forward for Kenyan litigation are as follows:

If the Kenyan courts rule, in the latter half of 2025, for the KPL (i.e., that the KPL can relitigate various legal issues), the Kenyan Courts will then schedule appropriate procedures for evidence presentation, written briefs, appeal hearings, and oral arguments on the substantive underlying constitutional issues. If allowed, the precise timeline for this potential new KPL substantive litigation cannot be known as of May 2025. But most likely, this new substantive appeal could not be resolved prior to court terms in 2026. If the Kenyan courts were to allow a substantive constitutional challenge about agricultural biotechnology, and if the Kenyan courts were to rule in favor of KPL’s constitutional challenge, then ultimately Kenyan courts would have reversed the decision of the E&L Court of 12 October 2023. In that event, due to litigation and judicial rulings, Kenya would then have a constitutional ban on agricultural biotechnology.

If the Kenyan courts rule against the KPL and uphold the ruling of the High Court C&HR, the Kenyan courts will have upheld the action of the Kenyan Government in October 2022 to lift a ban on genetically modified crops and foods. If the KPL 2025 relitigation appeal is denied, Kenya would have approved Bt cotton and Bt maize for cultivation and approved imports of genetically modified foods in accordance with the E&L Court decision. As the E&L Court stated, paraphrasing, as long as plant breeders comply with the regulatory structures (regulatory barriers) existing in Kenya to gain approval for genetically modified (and by implication gene-edited) crops and foods, Kenyan agriculture would be ready to begin using crops and foods created by modern plant breeding techniques.

But which path Kenya actually follows cannot be known in May 2025 because the chosen path depends on the outcome of the KPL 2025 relitigation appeal and potential future constitutional litigation likely extending into the year 2026. Hence, any prediction that Kenya appears to be on the verge of becoming a nation that consumes and grows genetically modified and gene-edited foods and crops has turned out, because of on-going litigation, to be premature [USDA GAIN Kenya, 2024, *passim*].

### Ghana

In January 2015, the Government of Ghana, through the National Biosafety Committee and the Ministry of Food and Agriculture, took steps to authorize the commercial release of Bt cowpea. In response to these steps, on 8 February 2015, Food Sovereignty of Ghana (FSG) (and others) filed a lawsuit to challenge the introduction of genetically modified crops into the nation. In this February 2015 legal challenge, FSG sought an injunction against the government. [Black’s Law Dictionary] In October 2015, the Accra High Court dismissed FSG’s application for an injunction (Ghana New Agency, 2015).

In response to this procedural loss, FSG filed a new lawsuit, on 23 November 2017, against the National Biosafety Committee and other governmental entities. FSG argued that the plans to commercialize genetically modified crops into Ghana had not followed authorizing legislation nor followed required administrative procedures (Gakpo, 2017). After several preliminary legal rulings, this 2017 lawsuit gave rise to the 24 May 2024 decision of the High Court of Human Rights (HCHR) in Accra: *Food Sovereignty of Ghana v. National Biosafety Authority* (NBA, 2024).

On 24 May 2024, the HCHR issued its opinion in the 9-year long litigation. In the opinion, the presiding Justice of the HCHR described the lawsuit as a “red herring” that had failed to provide evidence that undermined the Ghanaian regulatory system. The HCHR emphasized that the NBA and other agencies had conducted an extensive scientific and socio-economic review of the application for environmental release of genetically modified Bt cowpea. The HCHR ruled that the NBA and other agencies had complied with the Ghanaian Biosafety Act of 2011 and with international best practices. However, the Justice also directed the NBA to provide further public education and to finalize regulations related to labeling of genetically modified products before any commercial release of approved crops into Ghanaian farmers’ fields (Biotech Updates, 2024; NBA, 2024).

The HCHR decision upheld the NBA’s 2022 approval of the environmental release of Bt cowpea. While the HCHR 2024 decision indicated that Ghanaian governmental agencies have additional tasks to complete before the actual release of genetically modified crops, Ghana appears to be positioned to allow its farmers to grow genetically modified crops beginning in 2025. The author also thinks that the HCHR judgment impliedly affirmed the NBA’s decision, in April 2024, allowing the import of genetically modified maize and genetically modified soybean for food, feed and processing. Ghana imports significant amounts of foodstuffs and feedstuffs that contain processed genetically modified ingredients. Moreover, in 2023, the NBA adopted guidelines on gene-edited crops to provide clarity about which gene-edited crops are covered by the Biosafety Act of 2011 and which are not [USDA GAIN Ghana, 2024, *passim*.].

Taking into account the HCHR’s judgment and the NBA actions and decisions related to genetically modified crops and gene-edited crops, Ghana is moving forward to use modern plant breeding techniques in agriculture.

### The Philippines

MASIPAG, Greenpeace and other Filipino environmental organizations and individuals have been contesting governmental actions related to genetically modified crops in the Philippines since 2002. In 2002, the Philippines agricultural research universities and institutes began field testing Bt corn. In 2005, the Philippines government approved the commercial release of Bt. corn. Bt corn has been grown ever since in the Philippines. In 2012, as the Philippines government moved to approve field testing of Bt eggplant (talong in Tagalog), MASIPAG, Greenpeace, and their supporting complainants challenged the legality of these Bt eggplant field trials (Ocampo, 2013). In 2017, MASIPAG and Greenpeace similarly challenged applications for field trials and direct use of Golden Rice.

After many legal proceedings from 2012 through 2023, the Supreme Court of the Philippines consolidated the challenges to Bt eggplant and Golden Rice into a legal proceeding for the issuance of a Writ of Kalikasan. (In Tagalog, the word kalikasan means “nature” – Writ of Nature.) [Writ of Kalikasan defined] Therefore, a Writ of Kalikasan offers petitioners a legal procedure to seek legal protection for the environment.

On 18 April 2023, the Supreme Court of the Philippines, ordered the Court of Appeals Fourth Division in Manila to hear and resolve the consolidated lawsuit. [MASIPAG April 2024, pp. 2–7] Accordingly, the Court of Appeals issued two opinions in 2024 in this consolidated matter: *Magsasaka at Siyentipiko Para SA Pag-Unlad Agrikultura (MASIPAG) v. Secretary of Agriculture*, [MASIPAG April 2024] and *Magsasaka at Siyentipiko Para SA Pag-Unlad Agrikultura (MASIPAG) v. Secretary of Agriculture*, [MASIPAG August 2024]. MASIPAG August 2024 is an Amended Decision to the April 2024 decision.

The ruling of the Court of Appeals in August 2024 can best be stated by quoting from the decision itself.

p. 18 “The compelling interest and paramount importance of the instant case far outweigh technicalities that impede the cause of justice. If the stringent application thereof [procedural rules] would hinder rather than serve the demands of substantial justice, the former must yield to the latter…”

p. 19 “The precautionary principle applies; the grant of the privilege of the writs of kalikasan and continuing mandamus [supervision] [in this Court’s April 2024 decision] was proper. …

p. 20 There is no evidence that would show that GMOs do not pose greater risks than their conventional counterparts, especially considering that the [Secretary of Agriculture and other] regulators fell short of conducting the risk management and monitoring mechanisms required under the Joint Department Circulars (JDCs). Clearly, the determination of such risks can only be done if there are proper monitoring mechanisms implemented. Unfortunately, it was revealed during trial that the [Secretary of Agriculture and other] regulators have no such measures in place.

p. 20 All things considered the balancing of evidence adduced by the parties calls for a conclusion that the constitutional right of the people to a balanced and healthful ecology must be given the benefit of the doubt.” [MASIPAG August 2024, pages given in text quotations.]

Flowing from these rulings, the Court of Appeals ordered the Secretary of Agriculture and other respondent regulators to revoke the permits for the commercial release of Bt eggplant and Golden Rice, cease and desist from activities related to Bt eggplant and Golden Rice and (quoting):

p. 32 “Item 6. Enjoining the commercial propagation and/or conduct of activities relating to Golden Rice and Bt Eggplant until such time that the concerned respondent government agencies submit proof of safety and compliance with all legal requirements; and

“Item 7. Ordering concerned respondents government agencies to perform their mandate under the applicable JDC, by submitting to this Court the concrete mechanisms adopted to monitor all activities conducted under the JDCs, and all measures taken to strengthen the risk assessment procedure set forth in JDC No. 1–2021 in accordance with the ruling in this case.” [MASIPAG August 2024, pages given in text quotations.]

In the August 2024 amended decision, the Court of Appeals deleted Item 8 of its April 2024 Order. Item 8, from the April 2024 Order, had read as follows:

“Item 8. Enjoining any application for contained use, field testing, direct use as food or feed, or processing, commercial propagation, and importation of genetically modified organisms until compliance with (Item 7) [identical in both the April 2024 Order and the August 2024 order] above is established.” [MASIPAG April 2024, p. 142].

As written in April 2024, the Court by Item 8 had seemingly completely banned genetically modified crops and products, including imports, from the Philippines. But in the year 2024, the Philippines imported significant amounts of genetically modified food and feed. Moreover, the Philippines had been growing Bt maize for many years. Upon reconsideration of its April 2024 Order, the Court of Appeals in August 2024 ruled that other genetically modified crops and imports were not part of the MASIPAG case against Bt eggplant and Golden Rice. Hence, in August 2024, the Court Appeals decided that these other genetically modified crops and imports had not received an opportunity for being heard in litigation. Consequently, the Court of Appeals August 2024 Order deleted Item 8 of the April 2024 Order on the concern that Item 8 violated a fundamental right to due process. [MASIPAG August 2024, p. 31.]

The Philippine regulatory agencies have granted commercial release for four genetically modified crops: maize (2002), Golden Rice (2021), eggplant (2022), and cotton (2023). Filipino farmers planted 709,000 ha (1,751,939 acres) of genetically modified maize in the most recent planting year. The Philippines imports 60%–70% of its animal feed in the form of genetically modified maize or soybean. The Philippine regulatory agencies have also granted four certificates of non-genetically modified status to bananas and tomatoes created through gene-editing techniques. Filipino regulatory agencies are using “certificates of non-genetically modified status” to distinguish some gene edited crops from genetically modified crops [USDA GAIN Philippines, 2024, *passim*.].

The present status of this wide-spread use of genetically modified organisms in the Philippines is apparently the following:

The Court of Appeals ruling of August 2024 only applies to Golden Rice and Bt eggplant. As of May 2025, this Court of Appeals August 2024 ruling is apparently stayed (in abeyance) as the Philippine Rice Research Institute (PhilRice) appeals to the Supreme Court of the Philippines to reverse or modify the Court of Appeals decision (PhilRice, 2024). If the Supreme Court rules for PhilRice, Filipino farmers will have access to Golden Rice, Bt eggplant, Bt/HT maize, and Bt/HT cotton. If the Supreme Court rules against PhilRice, MASIPAG has successfully blocked Golden Rice and Bt Eggplant. Furthermore, MASIPAG would have a precedential (binding) decision upon which to rely to block genetically modified maize and cotton. MASIPAG would only need to file another lawsuit seeking a Writ of Kalikasan for these two additional crops.

As for imported genetically modified feedstuffs, the Court of Appeals August 2024 ruling allows continued importation, but MASIPAG threatens to file legal challenges against these imports [MASIPAG, 2024, pp. 2–3].

Concerning gene-edited crops, as contrasted with the more traditional genetically modified crops mentioned previously, the Court of Appeals decisions did not address these breeding techniques. The Court of Appeals decisions said nothing about the granting of certification of non-genetically modified status to bananas and tomatoes. MASIPAG likely opposes gene-edited crops as strongly as it opposes genetically modified crops and, therefore, seems likely to take legal action against gene-edited crops also. Thus, the ultimate legal status of gene-edited crops in the Philippine courts is an unknown, awaiting further legal proceedings and developments.

With litigation still in the Philippine Supreme Court, as of May 2025, the future of agricultural biotechnology is at a historical tipping point. If the Supreme Court rules for the Secretary of Agriculture, the Philippines could be an Asian leader in the use of biotechnology in agriculture. If the Supreme Court rules for MASIPAG, Filipino agriculture will be blocked from using agricultural biotechnology–and blocked for an indeterminate time into the future. Until this litigation is resolved, agriculture in the Philippines faces great uncertainty about the use or non-use of agricultural biotechnology.

### South Africa

In July of 2014, Monsanto Corporation applied for a permit for the general release of a drought-tolerant genetically modified maize (MON 87460). In 2015, the African Center for Biodiversity (ACB) opposed this application, thereby initiating litigation that lasted 9 years [ACB, 2023, ¶¶5–6]. The litigation resulted in two published opinions in the courts of South Africa: *African Centre for Biodiversity NPC against the Minister of Agriculture Forestry and Fisheries* (ACB, 2023), and *African Centre for Biodiversity NPC v. Minister of Agriculture, Forestry and Fisheries and others* (ACB, 2024).

In ACB 2023, the High Court denied the petition of the ACB and ruled as valid the general release of MON 87460. More specifically, the High Court judge wrote:

“[48] The Appeals Board considered everything that was before the EC (Executive Council for Genetically Modified Organisms) and had available the submissions of ACB, including the reports of their experts. The Appeal Board considered all of this and concluded that the permit should be granted. There is no evidence that the decision was either irrational or unreasonable. Nor was there any credible evidence that either the EC or the Appeals Board did not apply their minds to the information before them. Despite the allegations of bias and/or influence by third parties, no objective evidence was provided to prove that, or any unlawful delegation of power.” [ACB, 2023, ¶ cited in the text quotation.]

ACB appealed this High Court ruling to the Supreme Court of South Africa. The Supreme Court of South African set aside the High Court ruling and issued a new order. The Supreme Court ruled that the permit granted for MON 87460 was invalid and referred the Monsanto (now Bayer) application back to the regulatory agencies of South Africa for reconsideration [ACB, 2024, Order at ¶ 25(3)].

In ruling for the ACB, the Supreme Court wrote:

“[24] The high court conflated the obligation arising from section 5(1)(a) of the Act (Genetically Modified Organisms Act 15 of 1997) with the applicability of the precautionary principle, finding that an environmental impact study would only be required in the event of the precautionary principle being triggered. First, as addressed above, the precautionary principle was triggered and ought to have been applied. Second, whether the Executive Council, as a matter of fact, complied with section 5(1)(a) by considering the necessity of an environmental impact study to ascertain the impact on the environment of the proposed general release of MON 87460 was a separate and distinct inquiry from whether the precautionary principle was triggered and should have been applied. … The ineluctable conclusion is that the Executive Council failed to comply with a mandatory statutory prescript contained in section 5(1)(a). This means that the Executive Council’s decision cannot stand. Nor, for that matter, it must follow, can the decisions by the Appeal Board and the Minister.” [ACB, 2024, ¶ cited in the text quotation.]

South Africa has been growing genetically modified crops since the late 1990s. South Africa has approved thirty-three genetically modified traits for general release for cultivation [USDA GAIN, 2024, Table A1]. Since 2018, South Africa has approved field trials for an additional thirty-five genetically modified crops. [USDA GAIN, Table A2]. South African farmers widely plant three genetically modified crops: maize (corn), soybean, and cotton. As an example, for maize, South African farmers have used genetically modified varieties on more than 80% of planted hectares for the past seven crop years. [USDA GAIN, Table 1] South Africa has approved 108 genetically modified traits for import for food and feed use. [USDA GAIN, Table A3] South Africa is a exporter of genetically modified maize and soybeans [USDA GAIN, Table 2 &3]. South African agricultural research institutions are active in using modern biotechnology for crop breeding and improvement. [USDA GAIN, pp. 4–5]. Up until 2025, South Africa has been a world-wide leader in the adoption of genetically modified crops for cultivation, food, feed, import and export.

Although the Supreme Court of South Africa ACB 2024 decision is only about the general release of one genetically modified maize trait (MON 87640), the Supreme Court decision mandating both a distinct environmental impact statement and the application of the precautionary principle appears to apply to all genetically modified crops, field trials, foodstuffs and feedstuffs. Moreover, South African authorities ruled in August 2023 that new breeding techniques (for example, gene-editing techniques such as CRISPR, ZFN, and TALENS) will be regulated in the same manner as genetically modified organisms. [USDA GAIN, pp. 23–24] Thus, the Supreme Court ACB 2024 decision apparently will apply also to gene-edited crops, field trials, foodstuffs and feedstuffs.

South African agriculture is facing a very uncertain future regarding agricultural biotechnology. South Africa may change from being a world-leader in modern agricultural biotechnology to being a nation that, as a practical matter, bans it. South Africa may go from reaping the benefits of agricultural biotechnology to a nation that has chosen, through judicial decision, to abandon these benefits and to forego these benefits in the future (Cooper, 2022; Gbashi et al., 2021; Ala-Kokko et al., 2021; Gouse et al., 2016).

### United States

The United States Department of Agriculture, Animal and Plant Health Inspection Service (APHIS) first issued regulations for genetically modified plants in 1987. The 1987 regulations focused on risks from plant pests being used to genetically modify plants–most prominently *Agrobacterium tumefaciens* (a bacterium used to physically transfer a gene into a cell of the plant being transformed) and the use of promoter sequences from a plant virus (e.g., cauliflower mosaic virus). By the early years of 2000, plant breeders were able to genetically modify plants without the use of plant pests, either by direct gene transfer using “gene guns,” or by the removal of plant pest genes from the finished modified plant (null segregant plants), or through gene-edited techniques such as ZFN and TALENS. APHIS responded to non-pest modified plants by issuing letters to plant breeders, when plant breeders inquired, informing them that APHIS did not consider these non-pest modified plants to be within the APHIS regulations (Entine et al., 2021).

In a related development, the US government enacted the Plant Protection Act (PPA 2000), The PPA 2000 consolidated ten federal statutes dealing with plant pests, noxious weeds, invasive species and other potential threats to American agriculture. The PPA 2000 redefined noxious weed concerns as a consideration for APHIS in its regulatory mandate (APHIS, 2002).

Beginning in 2004, APHIS undertook a rule-making process to revise the US regulations governing plant biotechnology, taking into account PPA 2000, several executive orders, and other studies related to agricultural biotechnology regulation (Entine et al., 2021). APHIS completed this revision in 2020 with the adoption of a regulation (called the SECURE rule) that put more focus on the specific trait in the biotech plant, than upon the process by which the plant was created. [NFFC 2024, pp. 3–5]. Led by the National Family Farm Coalition and the Center for Food Safety, various opponents of agricultural biotechnology sued in the U.S. District Court for the Northern District of California asking for relief declaring the APHIS 2020 regulation invalid. Two published opinions resulted from this litigation: *National Family Farm Coalition v. Vilsack* (NFFC, 2024), and *National Family Farm Coalition v. Vilsack*, (NFFC, 2025).

The District Court Judge ruled in favor of the NFFC and its co-plaintiffs on two grounds. First, the District Court determined that APHIS had not adequately explained why it had not incorporated noxious weed considerations into its 2020 regulation [NFFC, 2024, p. 7–9]. Second, the District Court concluded that APHIS had not adequately explained why it exempted plants created by biotechnology from the 2020 regulation when these plants were equivalent to conventionally bred plants, even though conventionally bred plants can create, at times, plant pest and noxious week risks [NFFC, 2024, pp. 9–10]. The District Court vacated the 2020 regulation and remanded to APHIS for reconsideration of appropriate plant biotechnology regulations in a manner consistent with its 2024 opinion [NFFC, 2024, p. 2].

The District Court then addressed the appropriate remedy and wrote:

“…the Court is mindful that the rule took effect in 2020 and that this area of our national agricultural economy is rapidly developing. … at least 99 new GE plants have been exempted … retroactive vacatur ‘seems an invitation to chaos.’… [Black’s Law Dictionary].

“Consequently, the Court concludes that the remedy that best balances the law and that which ‘equity demands’ … is vacatur of the final rule as of the date of this order … this form of vacatur suffices to return the industry and GE-crop regulations to the *status quo ante*.” [NFFC, 2024, p. 13]

By vacating the rule “as of the date of this order,” the District Court was allowing previous approvals by APHIS under the 2020 SECURE regulation to remain valid, but also was telling APHIS that the 2020 SECURE regulation could no longer be used in future regulatory decisions.

As for the January 2025 opinion, the District Court in December 2024 told the parties to return to the Court to litigate unresolved issues. When the parties returned, the District Court decided that the unresolved issues were legally moot–i.e., no longer before the Court–because the December 2024 vacatur had settled all the issues. [NFCC, 2025, pp. 1–2]. In April 2025, the US dismissed its appeal from the District Court rulings. Hence, as of May 2025, APHIS regulates under the regulations written prior to 2020.

United States agricultural researchers extensively utilize biotechnology breeding techniques. United Sates farmers plant millions of hectares of genetically modified and gene-edited crops every crop year. Companies use genetically modified and gene-edited crops and ingredients from those crops in many foodstuffs and feedstuffs. American grocery shoppers consume biotechnology foodstuffs in great quantities. Animal agriculture in the US relies heavily upon genetically modified and gene-edited feedstuffs. The United States is the world’s leader in the adoption of biotechnology in agriculture.

The District Court opinions mean that APHIS has returned to a plant biotechnology rule that focused heavily upon the process by which plants were created. The APHIS regulations prior to 2020 used the trigger of genetic modification to assert regulatory control over plant biotechnology. How significant this return to “process” over “product” in agricultural biotechnology will be depends upon how APHIS responds. Even under the pre-2020 regulatory regime, US agriculture widely used genetically modified breeding techniques and crops. Thus, returning to the pre-2020 regulations does not mean a turn away from agricultural biotechnology for American agriculture. What the return to the pre-2020 regulations might mean is that the pace of the adoption of agricultural biotechnology may be slowed temporarily or may be slowed for a significant period of time into the future (Kershen and Miller, 2025).

## Part Two: commentary and critique

After reading the summaries of the judicial opinions of the seven jurisdictions, a reader could conclude that the opinions simply express the laws and regulations of that jurisdiction. While the reader might wish that the judicial opinions had been decided differently, the reader could think that the judges, who wrote those opinions, have done their best to understand and to interpret the facts and the laws in each particular legal matter. While this view of these opinions has validity, this benign acceptance of these opinions overlooks that the judges in each legal matter made choices about the facts and laws. The judges made choices about which legal interpretations to adopt as the rulings and orders of the court. The judges chose between competing, plausible interpretations of the laws and regulations of these seven jurisdictions.

The judges did not simply discover the facts and laws; the judges choose the facts and laws to reach their judicial conclusions. These judicial opinions reflect choices by human actors (judges). On the choices these judges made, this author can provide commentary and express critiques that are more than the personal fulminations of a pro-agricultural biotechnology author. By carefully reading the facts, laws, and interpretations of these judicial opinions, the author can highlight common themes. By carefully reading the facts, laws, and interpretations in these judicial opinions, the author can identify critiques based on the internal evidence of the lawsuits themselves. By so doing, the author’s commentary and critiques present an analysis of these legal matters that provides an understanding that the judges could have chosen differently. And, the author contends, the judges could have chosen better legal paths forward for agricultural biotechnology and for society as a whole.

### Agricultural science and innovation

The judicial opinions of New Zealand and the European Union (EU) are remarkably similar. In both opinions, the judges choose to subject newer, innovative gene-editing breeding techniques to the same legal and regulatory regime as previous genetic-engineering techniques. By so doing, the judges greatly slowed gene-editing from being used in their agricultural sectors. As a consequence of these judicial opinions, neither New Zealand or the EU have allowed gene-edited field trials or gene-edited crops to enter into the mainstream of agricultural research and farmers’ fields. Moreover, as New Zealand and the European Union have been very reluctant to authorize the cultivation of crops from previous genetic-engineering techniques (none in New Zealand and one in the EU), by subjecting gene-edited crops to the same regulatory system as previous genetic-engineering techniques, the judges have effectively precluded gene-edited crops from the fields of New Zealand and the EU.

In both instances, governmental authorities (in New Zealand, the EPA’s Section 26 Committee; in the EU, Advocate General Bobek) had expressed and argued a clear determination that many gene-edited techniques, including those techniques factually at issue in the actual litigation, were equivalent to chemical mutagenesis. As both New Zealand HSNO 1996 and EU Directive 2001/18/EC expressly exempt chemical mutagenesis from the burdens and strictures of the regulatory regimes deployed against previous genetic engineering, equivalent gene-edited techniques would be an expansion or extension of chemical mutagenesis. Therefore, the equivalent gene-edited technique would not be new and novel--as the legislators had considered the previous, regulated genetic-engineering techniques. The judges in the New Zealand and EU lawsuits could have chosen, legitimately, to adopt this equivalence and, consequentially, this exemption for gene-edited breeding techniques. By refusing to choose this equivalence option, the judges showed reluctance and skepticism to science (modern molecular biology and modern plant breeding) and to agricultural innovation (Monaco, 2024).

Similarly, in the recent judicial opinion (NFCC, 2024) in the United States, the judge could have ruled for USDA-APHIS. APHIS had spent 16 years developing a 2020 SECURE regulation focusing on the product of agricultural biotechnology, rather than focusing on the process used in agricultural biotechnology, as the pre-2020 regulations had done. By failing to understand or acknowledge the product versus process distinction, the judge disregarded scientific advances in molecular biology and agricultural innovation (McHughen, 2016).

The US judge showed a reluctant attitude toward more precise, better understood, more predictable techniques of plant breeding. The US judge required APHIS to return to outmoded and scientifically unjustified process triggers. The judge expressed an inkling of understanding when the judge developed a case-made remedy that vacated the 2020 rule only prospectively, rather than retroactively, so as to protect numerous gene-edited plants APHIS has already approved under the 2020 SECURE regulation. But that specially designed remedy also indicates, impliedly, that the judge could have granted APHIS a period of time (e.g., 120 days) to explain more fully its decisions about the 2020 SECURE regulation. At the end of that period, the judge then could have decided if the APHIS 2020 rule accorded with transparency, reasonableness and considered rationality. Instead, the judge made a choice to endorse arguments by opponents of agricultural biotechnology that, in this author’s opinion, are based on misunderstanding, misinformation and obscurantism about science and agriculture. The US judge fully disapproved the 2020 SECURE rule and sent the US regulatory system for agricultural biotechnology back to the past.

### Regulatory agencies of agricultural biotechnology

Courts rightly should hold regulatory agencies accountable for their decisions. Courts are tasked properly with insuring that regulatory agencies comply with their enabling laws and regulations. However, courts should not undermine trust in regulatory agencies by endorsing claims by opponents of agricultural biotechnology that attack the integrity of regulatory agencies. As the pleadings and presentations from the litigation in the seven jurisdictions shows, opponents of agricultural biotechnology consistently impugn the integrity of regulatory agencies. [e.g., MASIPAG April 2024, pp. 32–72, Court summary of the evidence presented by the parties to the litigation.] Judges should be very careful that they do not adopt the opponents’ attitude of impugning the integrity of regulatory agencies.

For example, in the litigation from South Africa, the Judge in the High Court (the lower trial court in South Africa) wrote as follows:

“[2] … The crux of ACB’s case is that the respective decision makers accepted the date included in Monsanto’s application at face value and without ensuring that the necessary health and safety risk associated with MON 87460 had been properly and independently assessed” [ACB, 2023, ¶ cited in text quotation.]

The High Court pointed out that “It (MON 87460) has been approved for use in food, animal feed and environmental release in 17 countries, including the United States, the European Union, Korea and Japan.” [ACB, 2023, ¶ 6] The High Court wrote:

“[48] … Nor was there any credible evidence that either the EC or the Appeals Board did not apply their minds to the information before them. Despite the allegations of bias and/or influence by third parties, no objective evidence was provided to prove that, or any unlawful delegation of power.” [ACB, 2023, ¶ cited in the text quotation.]

The High Court ruled in support of the regulatory agencies of South Africa which had granted the permit for general release.

The Supreme Court of South Africa expressed a different attitude and approach to the South African regulatory agencies than the High Court. The Supreme Court of South Africa summarized the ACB contentions as:

“[10] The thrust of the appellant’s (ACB] case is that the State respondents accepted at face value, the claims made by Monsanto and failed to independently an critically evaluate Monsanto’s application to satisfy themselves that the health and safety risks associated with the general release of MON 87460 had been properly addressed. … Accordingly, so the contention proceeds: (a) The Executive Council accepted the data submitted by Monsanto without any consideration of the veracity, accuracy and completeness thereof; (b) the Appeal Board did not engage with the ground of appeal and the expert evidence, but simply rubber-stamped the decision made by the Executive Council; and, (c) the Minister further rubber-stamped the Appeal Board’s decision by way of a confirmation letter that furnished no reasons at all.” [ACB, 2024, ¶ cited in the text quotation.]

The Supreme Court reversed the High Court judgment and agreed with the ACB contentions. The Supreme Court vacated the South African regulatory agencies’ approval for the general release of MON 87460.

The South African High Court and the South African Supreme Court exhibited strikingly different trust in the South African regulatory agencies governing agricultural biotechnology.

In contrast to the opinion of the Supreme Court of South Africa, the courts of Kenya and Ghana have been explicit in their trust for their nations’ regulatory systems for agricultural biotechnology. As the Kenyan Judge of the Environment and Land Court wrote:

“[339] Let me end by stating that as a country, we need to trust the institutions that we have in place, and call them to order in the event they breach the law. …

“…

“[342] With all these institutions, we should be confident that our health and environment is in good hands. It cannot be true that they have all conspired to expose the rest of the population to the calamities alluded to in the Petition.” [Law Society, 2023, ¶¶ cited in text quotations.]

Regulatory agencies in countries around the world have considered applications related to agricultural biotechnology for 30 years. Regulatory agencies around the world have ruled favorably for the safety of agricultural biotechnology for human health and the environment. Borrowing language from the Kenyan Judge, “It cannot be true that they [the regulatory agencies of countries around the world] have all conspired to expose the rest of the population to the calamities alluded to in …” the pleadings and presentations of opponents of agricultural biotechnology. After 30 years of regulatory considerations, courts should show greater respect and trust for the regulatory agencies of agricultural biotechnology.

### Precautionary principle

In the Philippines, the Supreme Court rules on the Writ of Kalikasan Rule 20 Precautionary Principle reads as follows:

“Section 1. Applicability – When there is a lack of full scientific certainty in establishing a causal link between human activity and environmental effect, the court shall apply the precautionary principle in resolving the case before it. The constitutional right of the people to a balanced and healthful ecology shall be given the benefit of the doubt.

“Section 2. Standards for application – In applying the precautionary principle, the following factors, among others, may be considered: (1) threats to human life or health; (2) inequity to present or future generations; or (3) prejudice to the environment without legal consideration of the environmental rights of those affected.” [MASIPAG April 2024, pp. 79–80]

The Court of Appeals referred to Rule 20 of the Writ of Kalikasan in stating:

“The precautionary principle bridges the gap in cases where scientific certainty in factual findings cannot be achieved. By applying the precautionary principle, the court may construe a set of facts as warranting either judicial action or inaction, to preserve and protect the environment. In effect, the precautionary principle shifts the burden of evidence of harm away from those likely to suffer damage and onto those desiring to change the *status quo*. Applying the precautionary principle to the rules of evidence will enable courts to tackle future environmental problems before ironclad scientific consensus emerges.” [MASIPAG April 2024, p. 80]

In this author’s opinion, using this understanding of the precautionary principle, the Court of Appeals thereby gave credence to MASIPAG and Greenpeace that an “ironclad scientific consensus” does not exist. As their opposition to agricultural biotechnology since 2002 manifests, MASIPAG and Greenpeace will never accept the overwhelming scientific evidence that genetically modified and gene-edited breeding techniques and crops present no new or different risks to human health or the environment than conventional breeding techniques and crops. It is worth recalling the words from a European Commission Report of 2010 stating:

“The main conclusion to be drawn from the efforts of more than 130 research projects, covering a period of more than 25 years of research and involving more than 500 independent research groups, is that biotechnology, and in particular GMOs, are not *per se* more risk than, for example, conventional plant breeding technologies.” (European Commission, 2010)

Thus, an overwhelming scientific consensus does exist of the risk-level (equivalent to conventional plant breeding) for genetic-modification as a technique. But the Court of Appeals, using its interpretation of the precautionary principle, did not consider this overwhelming scientific evidence to be “ironclad.”

Moreover, the Court of Appeals, using its interpretation of the precautionary principle, reversed the burden of presenting evidence and the burden of proof. Thus, the Court freed MASIPAG and Greenpeace from presenting evidence of harm from genetically modified crops (and there is no trustworthy evidence of meaningful harm to human health or the environment) and placed all the burden on the regulatory agencies to prove safety affirmatively, positively. Despite a fifteen-page summary of the regulatory agencies evidence about the safety of genetically modified crops for humans and the environment, the Court of Appeals evaluated this evidence as not sufficient to satisfy its interpretation of the precautionary principle’s demand for “ironclad scientific evidence.” [MASIPAG April 2024, pp. 16–31].

In refusing to consider the body of research to date as constituting ironclad scientific evidence of safety, the Court of Appeals, in effect, has positioned the Philippine judiciary as the ultimate arbiter of governmental decisions regarding agricultural biotechnology.

In the South African litigation, the Supreme Court quoted Principle 15 of the 1992 Rio Declaration on Environment and Development which states:

“The precautionary approach states that where there are threats of serious or irreversible damage, lack of full scientific certainty shall not be used as a reason for postponing cost-effective measures to prevent environmental degradation.” [ACB, 2024, ¶ 12]

Relying on Principle 15 of the Rio Declaration, the Supreme Court held:

“[18] The high court’s rejection of the appellant’s [ACB] reliance on the precautionary principle was based on its finding that the precautionary principle does not find direct application in the review proceedings. However, such an approach disregards the fundamental role that the precautionary principle plays in directing decision-makers in the exercise of their discretion. The current state of knowledge and uncertainty, the potential for serious or irreversible harm and the adoption of a cautious approach is clearly consistent with the subject-matter, scope and purpose of the [Biosafety] Act [of 1997].” [ACB, 2024, ¶ cited in text quotation.]

While the Supreme Court of South Africa quoted Principle 15 of the Rio Declaration, the Court passed over and did not quote other principles from the Rio Declaration. For example, Principle 1 which reads:

“Human beings are at the centre of concerns for sustainable development. They are entitled to a healthy and productive life in harmony with nature.” (United Nations, 1992)

Or, for example, Principle 5 which reads:

“All States and all people shall cooperate in the essential task of eradicating poverty as an indispensable requirement for sustainable development, in order to decrease the disparities in standards of living and better meet the needs of the majority of the people of the world.” (United Nations, 1992)

By only quoting Principle 15, the Supreme Court of South Africa made no attempt at considering or reconciling other Principles in the Rio Declaration that may cast a more favorable light upon the work and decisions of the South African agencies related to agricultural biotechnology.

By ruling that the precautionary principle of Principle 15 alone applied in the litigation, the Supreme Court of South Africa seemingly arrogated to itself vast powers over agricultural biotechnology. Like the Court of Appeals of the Philippines, the Supreme Court decision in ACB 2024 apparently positions the judiciary to be the ultimate arbiter of governmental decisions regarding agricultural biotechnology.

Philippine and South African courts have disregarded 30 years of experience with agricultural biotechnology in which not a single instance of serious or irreversible damage exists to either human health or the environment. The courts disregarded this 30-year experience both world-wide and in their own nations. Moreover, the courts did not acknowledge the considerable benefits for human health, the environment, and the agricultural sector that agricultural biotechnology offers. The regulatory agencies of the Philippines and South Africa had offered this evidence of lack of harm and of benefits in the voluminous information comprising the litigation record. Rather, the courts chose to adopt the perspective of MASIPAG, Greenpeace, and ACB that speculative hazards should be given preference to lived experience and proven benefits. By so doing, the courts did not allow the precautionary principle to be a principle of common sense. Rather, the courts allowed opponents of agricultural biotechnology to use the precautionary principle as a trump card to block biotechnology from the agricultural sector. Under the circumstances of these cases, this is an abuse of the precautionary principle [Commission Staff, 2021, passim; Purnhagen and Wesseler, 2025, passim; Tagliabue, 2016, passim].

### Value choices

In these legal proceedings, the opponents of agricultural biotechnology exhibit cynicism tied to their implacable opposition. From this author’s perspective, their cynicism is unmoored from facts, science, truth, and morality. Several examples from the litigation provide evidence of this cynicism.

In the litigation in Kenya, the petitioners (opponents of agricultural biotechnology) attacked the Executive Order of October 2022 lifting a ban on cultivation of agricultural biotechnology crops. The ban originated with a prior Executive Order issued on 12 November 2012 that had asserted concerns for human health as justification for the summary action. The Executive Order of 2012 based its concerns upon a 2012 published study by Séralini, E. and others [Law Society, 2023, ¶¶ 31–36 of Judgment]. Within days of the publication of the Séralini study, many scientists criticized the study for its methodology, its data interpretation, and its conclusions. By September and October of 2012, the controversy about the Séralini study was widely reported (Independent Science News, 2012; Mole, 2012). Thus, prior to the Executive Order 2012 in November 2012, the Kenyan Executive knew or should have known of the controversy about the Séralini study. As time passed, many scientists thoroughly refuted the Séralini study (FCT March, 2013; Arjó et al., 2013; Coumoul et al., 2018; Admin, 2018). The publisher retracted the study (Elsevier, 2013). In 2012, the Kenyan Executive summarily entered the 2012 Order without trusting the Kenyan regulatory agencies carefully to consider and assess the implications of the Séralini study for the regulation of agricultural biotechnology in Kenya. Rather, the Executive Order 2012 hobbled the Kenyan regulatory agencies making them relatively inactive for 10 years, until the Executive Order 2022, despite the valid criticisms and retraction of the Séralini study.

In the *Law Society* litigation, the petitioners opposed to agricultural biotechnology alleged that the Executive lifting of the ban in October 2022 was done illegally because it was done in a summary manner. The High Court E&L Judge rejected this allegation and remarked:

“[250] As to whether there was public participation before the Cabinet Dispatch of October 2022, I have not been shown any law or authority that requires the Cabinet to engage the public before arriving at its decisions. In any event, there was no public participation before the ban of GMOs was imposed by the Cabinet in 2012.” [Law Society, 2023, ¶ cited in the text quotation.]

As the quotation from the opinion of the High Court E&L clearly implies, the petitioners against the Executive Order 2022 were not opposed to summary action, but only summary action that did not align with their opposition to agricultural biotechnology and their opposition to a robustly functioning Kenyan regulatory system.

In August 2024, the Court of Appeal of the Philippines amended its April 2024 decision. The Court did so in response to motions for reconsideration. MASIPAG and Greenpeace requested reconsideration giving the following explanation to the Court of Appeals:

“The petitioners [MASIPAG and Greenpeace] pray that this Court modify Item (8) of the (of the April 2024 judgment) on the ground of newly discovered evidence. They submitted an online BusinessWorld Article … on 29 April 2024 entitled, “*How may the Philippines be affected by the Court of Appeals 2024 Writ of Kalikasan?*” explaining that the Philippines imports genetically modified organisms (GMOS) such as yellow corn and soya meal, which are the most important feed ingredients in animal feeds. Since these animal feeds comprise 60%–70% of the total cost of pork, poultry meat and egg production, the Court’s directive to ban GMO importation would have grave and adverse economic effect on the country’s swine and poultry industries.” [MASIPAG August 2024, p. 4]

The reader should note that the immediately preceding quotation is from a MASIPAG and Greenpeace document asking the Court of Appeals to reconsider Item 8 of the April 2024 order. As previously explained in the summary of the Filipino litigation, the Court of Appeal did decide to strike Item 8 from the final August 2024 Order. Why did MASIPAG and Greenpeace undermine their own implacable opposition to genetically modified crops, foods, and feeds in the Philippines by asking for the deletion of Item 8? One can properly ask: did MASIPAG and Greenpeace change their legal stance due to newly discovered evidence, as the quotation says? Information about the massive quantity of imported genetically modified corn and genetically modified soybean, as the feed supply for the livestock industry of the Philippines, was widely and easily known prior to the April 2024 Order. Or, one can properly ask: did MASIPAG and Greenpeace reconsider because of cynical political calculations about the likely societal uproar from Filipinos who, if Item 8 were imposed, would be deprived of meat supplies and be forced to pay more for the lessened supply? The record of the litigation provides no answer to these two “one-can-properly-ask” questions. What the record does show is that by their request for reconsideration to delete Item 8 from the final August 2024 Order, MASIPAG and Greenpeace were more concerned about feeding livestock than feeding Filipinos. By contrast, MASIPAG and Greenpeace sought and won a court order that deprived Filipinos especially subsistence farmers and the urban poor–those who would most benefit from Golden Rice and Bt eggplant, – of nutritious food (Wu et al., 2021). The cynicism of MASIPAG and Greenpeace, at least to this author, seems obvious: feeding livestock is preferred to feeding Filipino people.

The Court of Appeals did modify its April 2024 Order by deleting Item 8 from its August 2024 Order. The Court reasoned that the Item (8) ban on importation of food and feed raised significant due process concerns because the importers of these foodstuffs and feedstuffs were not parties to the litigation [MASIPAG August 2024, pp. 30–31].

However, when the Court of Appeals ruled for MASIPAG and Greenpeace to delete Item 8 in August 2024, the Court did so without discussing or even citing any constitutional provisions or statutes of the Philippines or any international standards that proclaim human welfare as a worthwhile and co-equal value [Dubock, 2024, *passim*]. The Court of Appeals did not seriously consider that the Writ of Kalikasan (the Writ of Nature) might not be an absolute, predominant right in the Philippines to the detriment of human welfare. The Court of Appeals simply gave no consideration to balancing co-equal values. By endorsing the ban on Golden Rice and Bt eggplant, the Court of Appeals endorsed the legal argument that the Filipino environment is preferred to human welfare of the Filipino people.

No wonder that by May 2025 179 Nobel Laureates have signed a letter, drafted in 2016, protesting Greenpeace and MASIPAG and their campaign against agricultural biotechnology, especially Golden Rice. Further, the Laureates called for governments and international governance organizations to reject these campaigns against agricultural biotechnology. As the Laureates state: “Opposition based on emotion and dogma contradicted by data must be stopped.” (Laureates Letter, 2016).

## Conclusion

History will judge whether the opponents or the proponents of agricultural biotechnology better protected human health and the environment. What future historians will write and conclude is unknown and unknowable in May 2025. What is known and predictable is that the pace of scientific discovery and the expansion of scientific knowledge, related to agricultural biotechnology, will not slow. Recent examples include a new gene-editing system from Chinese scientists (Chinese scientists, 2025), detailed plant genomes (Plant Genomes, 2025), gene-edited tilapia from Brazil (Brazilian fish, 2025), plant-based foods with animal genes from Argentina (Pork-Infused Soybeans, 2025), gene-transfer from hornworts (an algae) to food crops for CO2 efficiency (Funny Little Plant, 2025).

What can also be predicted is that groups that oppose agricultural science and innovation, that mistrust regulatory agencies regarding biotechnology, that employ the precautionary principle to ban breeding techniques and their products, and that place livestock and the environment (as an abstract entity, rather than actual environments) over the welfare of people will file lawsuits. If this survey of seven jurisdictions and litigation is any guide, these lawsuits may often find receptive judges who make choices about the facts, laws, regulations, and interpretations that deny these scientific achievements to farmers and plant scientists. This survey of litigation in seven jurisdictions presents clear evidence that judges in their choices can be significant impediments to the adoption of agricultural biotechnology. This survey also presents clear evidence that judges in their choices, alternatively, can meaningfully allow a way forward for agricultural biotechnology.

## Glossary

### Black’s Law Dictionary (8th edition, 2004) (Garner, B. Editor-in-Chief):

Collateral estoppel: 1. The binding effect of a judgment as to matters actually litigated and determined in one action on later controversies between the parties involving a different claim from that on which the original judgment was based. 2. A doctrine barring a party from relitigating an issue determined against that party in an earlier action, even if the second action differs significantly from the first one.
Injunction: A court order commanding or preventing an action. To get an injunction, the complainant must show that there is no plain, adequate, and complete remedy in law and that an irreparable injury will result unless the relief is granted.
Res judicata: 1. An issue that has been definitively settled by judicial decision. 2. An affirmative defense barring the same parties from litigating a second lawsuit on the same claim, or any other claim arising from the same transaction or series of transactions and that could have been – but was not – raised in the first suit.
Vacatur: 1. The act of annulling or setting aside. 2. A rule or order by which a proceeding is vacated.
Writ of Kalikasan: Writ of Kalikasan means a legal remedy available to any natural or juridical person, entity authorized by law, people's organization, non-governmental organization, or any public interest group accredited by or registered with any government agency, on behalf of persons whose constitutional right to a balanced and healthful ecology is violated, or threatened with violation by an unlawful act or omission of a public official or employee, or private individual or entity, involving environmental damage of such magnitude as to prejudice the life, health or property of inhabitants in two or more cities or provinces. (Rules of Procedure for Environmental Cases A.M. No. 09-6-8-SC Rule 7, Sec. 1) “Kalikasan” is a Filipino word which in English, means “Nature”. http://notocoal.weebly.com/writ-of-kalikasan.html.

## References

[B1] ACB (2023). African centre for biodiversity NPC against Minister of agriculture, forestry and fisheries, case no. 27524/2017, high court of South Africa, Gauteng division Pretoria (June 27, 2023).

[B2] ACB (2024). African centre of biodiversity NPC V. Minister of agriculture, forestry and fisheries and others (934/2023) ZASCA 143 (22 October 2024).

[B3] Admin S. (2018). EU’s Wholes Refutation of Seralini’s Anti-GMO ‘Science. Available online at: http://bioscriptionblog.com/2018/12/18/eu-refutation-seralini-anti-gmo .

[B4] Ala-Kokko K. Lawton L. Shew A. Tack J. Chaminuka P. Matlock M. D. (2021). Economic and ecosystem impacts of GM maize in South Africa. Glob. Food Secur. 29, 100544. 10.1016/j.gfs.2021.100544

[B5] APHIS (2002). APHIS factsheet: the plant protection act (June 2002). Available online at: https://www.govinfo.gov/content/pkg/GOVPUB-A101-PURL-LPS99695.pdf.

[B6] Arjó G. Portero M. Piñol C. Viñas J. Matias-Guiu X. Capell T. (2013). Plurality of opinion, scientific discourse and pseudoscience: an in depth analysis of the Séralini et al. study claiming that Roundup™ Ready corn or the herbicide Roundup™ cause cancer in rats. Transgenic Res. 22, 255–267. 10.1007/s11248-013-9692-9 23430588

[B7] Biotech Updates (2024). Landmark Ruling as Ghanaian Court Dismisses Suit Challenging GM, ISAAA Inc. Manila. Available online at: https://www.isaaa.org/kc/cropbiotechupdates/article/default.asp?ID=21843 .

[B8] Bobek (2018). Opinion of advocate general (bobek). Case C-52816, Confédération paysanne v. Prime Minist. Available online at: http://curia/europa.eu/juris/document.

[B9] Brazilian Fish (2025). Brazilian fish announces the first genetically edited tilapia for performance in Brazil. Press Release. Available online at: https://www.brazilianfish.com.br/news/brazilian-fish-anuncia-a-primeira-tilapia-geneticamente-editada-para-desempenho-no-brazil.

[B10] Brooks G. Smyth S. (2024). As the EU’s regulatory stance on gene-edited crops slips back toward “precautionary inertia,” in Europe’s over-precaution threatens global food security, Cincinnati OH: Genetic Literacy Project. Available online at: https://geneticliteracyproject.org/2024/08/01/as-the-eu-regulatory-stance-on-gene-edited-crops-slips-back-toward-precautionary-inertia-europes-over-precaution-threatens-global-food-security/.

[B11] Chinese Scientists (2025). Bela, V. Chinese scientists use dengue fever virus to build safer gene-editing system. South China Morning Post. Hong. Kong. Available online at: https://www.scmp.com/news/china/science/article/3298949/chinese-scientists-use-dengue-fever-virus-build-safer-gene-editing-system.

[B12] Commission Staff Working Document (2021). Study of the status of new genomic techniques under union law and in the light of the Court of Justice ruling in case C-528/16. European Commission. Available online at: https://food.ec.europa.eu/system/files/2021-04/gmo_mod-bio_ngt_eu-study.pdf.

[B13] Confédération paysanne (2015). Press release with timeline. Available online at: https://www.confedertionpaysanne.fr/actu.php?id=5762.

[B14] Confédération paysanne against Prime Minister (2018). Case C-528/16 (grand chamber, CJEU) (25 July 2018). Available online at: http://curia.europa.eu/juris/document.

[B15] Confédération paysanne against Prime Minister (2023). Case C-688/21 (grand chamber, CJEU). Available online at: http://curia.europa.eu/juris/document.

[B16] Cooper C. (2022). Evolution of *Bacillus thuringiensis* maize yields in South Africa, ScholarWorks@UARK (August 2022).

[B17] Coumoul X. Servien R. Juricek L. Kaddouch-Amar Y. Lippi Y. Berthelot L. (2018). The GMO90+ project: absence of evidence for biologically meaningful effects of genetically modified maize-based diets on wistar rats after 6-Months feeding comparative trial. Toxicol. Sci. 168, 315–338. 10.1093/toxsci/kfy298 PMC6432862 30535037

[B18] Dubock A. (2024). The sustainable development goals are unattainable without a change of nutritional strategy. Eur. Soc. Med. Med. Res. Archives 12 (8). 10.18103/mra.v12i10.5818

[B19] Elsevier Press Release (2013). Elsevier announces article retraction from journal of Food and Chemical Toxicology (Nov. 28, 2013).

[B20] Entine J. Felipe M. Groenewald J. Kershen D. Lema M. McHughen A. (2021). Regulatory approaches for genome edited agricultural plants in select countries and jurisdictions around the world. Tran. Res. 30, 551–584. 10.1007/s11248-021-00257-8 PMC8316157 33970411

[B21] European Commission (2010). A decade of EU-Funded GMO research (2001-2010). Available online at: https://ec.europa.eu/research/biosociety/pdf/a_decade_of_eu-funded_gmo_research.pdf.

[B22] FCT March (2013). Food and chemical toxicology, 53 (1), 440–483. 10.1016/j.fct.2012.10.055 23142679

[B23] Funny Little Plant (2025). Schmitt, K. The funny little plant that could upend agriculture. Available online at: https://ambrook.com/research.

[B24] Gakpo J. (2017). Ghana anti-GMO activists seek court ruling against improved crops. Available online at: https://allianceforscience.org/blog/2017/12/ghana-anti-gmo-activists-seek-court-ruling-against-improved-crops/.

[B77] Garner B. A. (2004). Black's Law Dictionary (8th Edition). Eagan, MN: Thomson West Publishing Co.

[B25] Gbashi S. Adebo O. Adebiyi J. Targuma S. Tebele S. Areo O. M. (2021). Food safety, food security and genetically modified organisms in Africa: a current perspective. Biotechnol. Genet. Eng. Rev. 37 (1), 30–63. 10.1080/02648725.2021.1940735 34309495

[B26] Gene Technology Bill (2024). Available online at: https://www.parliament.nz/en/pb/sc/make-a-submission/document/54SCHEA_SCF_22059628-B0CC-4931-5E07-08DD18A12BFB/gene-technology-bill.

[B27] Ghana News Agency (2015). Court dismisses GMO case in Ghana. Available online at: https://africenter.isaaa.org/court-dismisses-gmo-case-in-ghana.

[B28] Gouse M. Sengupta D. Zambrano P. Falck-Zepeda J. (2016). Genetically modified maize: less drudgery for her, more maize for him? Evidence from smallholder maize farmers in South Africa. World Dev. 83, 27–38. 10.1016/j.worlddev.2016.03.008

[B29] Independent Science News (2012). Séralini and science: an open letter. Available online at: https://www.independentsciencenews.org/health/seralini-and-science-nk603-rat-study-roundup.

[B30] Kenya Peasants League v. Attorney General (2025). Court of appeal at Nairobi, [2025] KFCA 448 (KLR).

[B31] Kershen D. (2015). Sustainability council of New Zealand trust v. The environmental protection authority: gene editing technologies and the law. GM Crops Food 6 (4), 216–222. 10.1080/21645698.2015.1122859 26618752 PMC5033166

[B32] Kershen D. Miller H. (2025). “After an unexpected and controversial federal ruling, the regulatory future of gene edited crops in the US is cloudy,” in Cincinnati OH: Genetic Literacy Project. Available online at: https://geneticliteracyproject.org/2025/02/24/viewpoint-after-an-unexpected-and-controversial-federal-ruling-the-regulatory-future-of-gene-edited-crops-is-suddenly-in-limbo/.

[B33] Laureates Letter (2016). Laureates letter supporting precision agriculture (GMOs). Available online at: https://supportprecisionagriculture.org/nobel-laureate-gmo-letter_rjr.html.

[B34] Law Society of Kenya (2023). Attorney general (environmental and planning petition 2), [2023] KEELC 20682 (KLR) (Oct. 12, 2023).

[B35] Magsasaka at Siyentipiko Para SA Pag-Unlad Agrikultura (MASIPAG) v.Secretary of agriculture, CA-G.R. SP no. 00038 (2024b). [MASIPAG August 2024].

[B36] Magsasaka at Siyentipiko Para SA Pag-Unlad Agrikultura (MASIPAG) v.Secretary of agriculture, CA-G.R. SPP no. 00038 (2024a). [MASIPAG April 2024].

[B37] McHughen A. (2016). A critical assessment of regulatory triggers for products of biotechnology: product vs. process. GM Crops Food 7, 125–158. 10.1080/21645698.2016.1228516 27813691 PMC5161003

[B38] Mole B. (2012). *GM crop concerns*, The Scientist (September 20, 2012).

[B39] Monaco A. (2024). The role of heuristics and biases in the choice of risk triggers for novel foods and GMOS in the European Union. Eur. J. Risk Regul. 16 (2024), 217–227. 10.1017/err.2024.48

[B40] Morrison Forester (2025). Client alert: update on regulation of new genomic techniques in the EU. Available online at: https://www.mofo.com/resources/insights/250425-april-2025-update-on-regulation.

[B41] Mwangi v. Attorney General (2024). [2024] KEHC 13678 (KLR).

[B42] National Biosafety Authority (NBA-Ghana) (2024). Press release: human rights court dismisses food sovereignty suit against NBA over commercialisation of GM products in Ghana (24 May 2024).

[B43] Negri M. (2024). *Vandals destroy Italy’s first experimental rice field, Reuters* (June 24, 2024). Italian scientists used CRISPR/Cas9 to breed the rice.

[B44] NFFC (2024). National family farm coalition v. Vilsack, 21-cv-05695-JD (N.D. Cal., December 2, 2024 slip opinion issued by the Court).

[B45] NFFC (2025). National family farm coalition v. Vilsack, 21-cv-05695-JD (N.D. Cal., January 28, 2025 slip opinion issued by the Court).

[B46] Ocampo S. (2013). Opinion: bt talong field testing: a boon or health threat? The Philippine Star (June 29, 2013) (discussion of opposition to genetically modified crops from 2002 through 2013).

[B47] Philippine Rice Research Institute (2024). Press Release: Philrice seeks reconsideration of CA ruling on Malusog Rice. Available online at: https://www.philrice.gov.ph/philrice-seeks-reconsideration-of-ca-ruling-on-malusog-rice/.

[B48] Plant Genomes (2025). Gerhard, D., Plant genomics is blooming, and it could change how we grow food, The Scientist (February 20, 2025).

[B49] Pork-Infused Soybeans (2025). Watkins, K., pork-infused soybeans and beef-infused peas. Available online at: https://ambrook.com/research.

[B50] Purnhagen K. Wesseler J. (2025). “Precaution and the precautionary principle: a view on the EU – the example of modern biotechnology,” in Encyclopedia of law and economics. 10.1007/978-1-4614-6_835-1

[B51] Szpunar (2022). Opinion of Advocate General (Szpunar), Case C-688/21 Confédération paysanne v. Prime Minist. Available online at: http://curia.europa.eu/juris/document.

[B52] Tabliabue G. (2016). The Precautionary principle: its misunderstandings and misuses in relation to GMOs. New Biotechnol. 33 (4), 437–439. 10.1016/j.nbt.2016.02.007 26923807

[B53] The Sustainability Council of New Zealand Trust v. The Environmental Protection Authority (2014) NZHC 1067. 10.1080/21645698.2015.1122859 PMC5033166 26618752

[B54] United Nations (1992). Report of the United Nations Conference on environment and development. Annex I – Rio Declar. of Environ. Dev. Available online at: http://www.un.org/documents/ga/conf151/aconf15126-1annex1.htm.

[B55] USDA-GAIN (2023). Biotechnology and other new production technologies annual: European Union, Report Number: E42023-0047 (Dec. 8, 2023).

[B56] USDA-GAIN (2023). Agricultural biotechnology annual: New Zealand, Report Number: NZ2023-0015 (Nov. 27, 2023).

[B57] USDA-GAIN (2024). Agricultural biotechnology annual: Ghana, Report Number: GH2024-0012. (Nov. 4, 2024)

[B58] USDA-GAIN (2024). Agricultural biotechnology annual: Kenya, Report Number: KE2024-0011 (Nov. 24, 2024).

[B59] USDA-GAIN (2024). Agricultural biotechnology annual: Philippines, Report Number: RP2024-0039 (Nov. 12, 2024).

[B60] USDA-GAIN (2024). Agricultural biotechnology annual: South Africa, Republic of, Report Number: SF2024-0027 (November 20, 2024).

[B61] Wu F. Wessler J. Zilberman D. Russel R. Chen C. Dubock A. (2021). Opinion: Allow Golden Rice to save lives. Proc. Nat. Acad. Sci. U. S. A. 118 (51), e2120901118. 10.1073/pnas.2120901118 PMC8713968 34911769

